# CXCL9 associates with experimental neuromyelitis optica spectrum disorder following adoptive transfer of Tfh and Th17 cells

**DOI:** 10.3389/fimmu.2026.1704040

**Published:** 2026-05-28

**Authors:** Liang Wang, Lei Zhou, Wenjuan Huang, Jingzi ZhangBao, Hongmei Tan, Yuxin Fan, Chuanzhen Lu, Jian Yu, Min Wang, Jiahong Lu, Chongbo Zhao, Jun Wang, Chao Quan

**Affiliations:** 1 Department of Neurology and Rare Disease Center, Huashan Hospital, Shanghai Medical College, Fudan University, Shanghai, China; 2 National Center for Neurological Disorders, Shanghai, China; 3 Department of Ophthalmology and Vision Science, Eye and ENT Hospital, Shanghai Medical College, Fudan University, Shanghai, China; 4 Department of Integrative Medicine and Neurobiology, School of Basic Medical Science, Shanghai Medical College, Shanghai Key Laboratory of Acupuncture Mechanism and Acupoint Function, Fudan University, Shanghai, China

**Keywords:** aquaporin-4, CXCL9, follicular helper T cell, neuromyelitis optica spectrum disorder, T helper 17 cell

## Abstract

**Background:**

This study investigates the pathogenic contributions of aquaporin-4 (AQP4)-specific follicular helper T (Tfh) and T helper 17 (Th17) cells in neuromyelitis optica spectrum disorder (NMOSD), utilizing newly established murine models based on adoptive transfer of antigen-specific T-cell populations.

**Methods:**

AQP4-knockout mice were immunized with the AQP4-derived peptide to generate AQP4-reactive Tfh and Th17 cells. These cells were subsequently isolated and adoptively transferred into wild-type recipient mice. At disease peak—defined by consistent neurological deficits—spinal cord and brain tissues were harvested for histopathological analysis, as well as immunohistochemistry. Central nervous system immune cell infiltration was quantified via flow cytometry. Total RNA was extracted from spinal cord tissue for bulk RNA sequencing; differentially expressed genes were validated using quantitative real-time PCR.

**Results:**

Recipient mice that received AQP4-reactive Tfh or Th17 cells developed progressive hind-limb weakness, with Th17-transferred mice exhibiting significantly more severe clinical scores. Histopathological analyses revealed robust perivascular inflammation, parenchymal immune infiltration, and focal demyelination. Immunohistochemical quantification demonstrated significantly increased the optical density of CD3, B220, GFAP, IBA1, and CXCL9, alongside markedly decreased MBP expression. Flow cytometric profiling confirmed substantial infiltration of leukocytes and activated microglia/macrophages into the central nervous system (CNS). Transcriptomic analysis identified CXCL9 as one of the most upregulated chemokines in the spinal cord; its astrocytic origin was further corroborated by confocal immunofluorescence co-localization with GFAP.

**Conclusion:**

Our findings establish that AQP4-specific Tfh and Th17 cells are sufficient to drive key neuropathological features of NMOSD—including microglial reactivity, leukocyte recruitment, neuroinflammation, and demyelination—*in vivo*. The pronounced upregulation and astrocyte-derived expression of CXCL9 suggest its involvement in orchestrating CNS inflammation and position it as a potential contributor for NMOSD.

## 1 Introduction

Neuromyelitis optica spectrum disorder (NMOSD) is a heterogeneous autoimmune disease characterized by severe neuroinflammation, astrocyte injury, and secondary demyelination, leading to debilitating motor, visual, and autonomic neurological deficits (1). Pathogenic immunoglobulin G autoantibodies against aquaporin-4 (AQP4-ab) are detected in approximately 75%-80% of NMOSD patients and constitute a defining serological biomarker with direct pathogenic relevance (2–4). AQP4—highly enriched on astrocytic end-feet at the blood–brain barrier—represents a well-validated immunological target, thereby providing a mechanistically grounded foundation for developing preclinical models to dissect disease pathogenesis and identify novel therapeutic interventions.

An ideal animal model of AQP4-ab-positive NMOSD should faithfully recapitulate hallmark neuropathological features: (i) primary astrocytopathy, evidenced by selective loss of AQP4 and glial fibrillary acidic protein (GFAP); (ii) secondary oligodendrocyte and neuronal damage; and (iii) robust central nervous system (CNS) infiltration by macrophages/microglia, neutrophils, eosinophils, and T and B lymphocytes, alongside complement activation and deposition (5). While actively immunized models (e.g., peptide- or protein-adjuvant immunization) better mimic chronic immune sensitization and disease evolution, passively immunized models (e.g., intrathecal or systemic AQP4-ab administration) enable precise dissection of antibody-mediated effector mechanisms. Current preclinical strategies for modeling AQP4-ab-positive NMOSD include (i) AQP4-ab/complement co-injection in experimental autoimmune encephalomyelitis (EAE)-sensitized mice; (ii) direct intracerebral or intraspinal AQP4-ab/complement delivery; (iii) cytokine-induced disruption of blood–brain barrier integrity; and (iv) adoptive transfer of AQP4-specific CD4^+^T cells (6–8). Despite these advances, the distinct contributions of AQP4-specific follicular helper T (Tfh) and T helper 17 (Th17) cells—two functionally specialized CD4^+^T-cell subsets critically involved in B-cell help and tissue inflammation—remain incompletely characterized in NMOSD pathogenesis.

Passive transfer of AQP4-reactive Th17 cells into wild-type recipients has been described previously as a model of NMOSD. In this study, we extended this approach by establishing, for the first time, an adoptive transfer model using AQP4-reactive Tfh cells, as Tfh cells were clarified to play a critical role in the pathogenesis of AQP4-ab-positive NMOSD (9). By comparing Tfh- and Th17-driven pathology, we aimed to identify subset-specific transcriptomic signatures. Our main focus is on the discovery that CXCL9 is strongly associated with the Tfh-mediated inflammatory response, which we validated in both models, primary astrocytes after intervention with AQP4-ab and human samples. Thus, the key novelty of this work lies not in the Th17 transfer technique per se but in the new Tfh transfer model and the CXCL9-related findings that shed light on differential T-cell subset contributions in NMOSD.

## 2 Materials and methods

### 2.1 Animals and human tissue samples

AQP4-knockout (AQP4-KO) mice on a C57BL/6 background were obtained (Cyagen Biosciences, China) and maintained under specific pathogen-free conditions in the institutional animal facility. Female wild-type C57BL/6 mice (6–8 weeks of age) were purchased (Shanghai Jiesijie Laboratory Animal, China). All mice had ad libitum access to autoclaved food and acidified water and were acclimatized for at least 3 days prior to experimentation. Human brain tissue was obtained postmortem from a histologically confirmed AQP4-ab-positive NMOSD patient who had undergone neurosurgical resection for an incidental supratentorial mass (diagnosed as grade II oligodendroglioma); no clinical or radiological evidence of active NMOSD lesions was present at the time of surgery. Written informed consent for tissue donation and research use was obtained from the patient prior to surgery. The study protocol involving human tissue were reviewed and approved by the Ethics Committee of Huashan Hospital, Fudan University, and conducted in strict accordance with the Declaration of Helsinki and the guidelines of the International Council for Laboratory Animal Science.

### 2.2 Induction and clinical assessment of experimental NMOSD models

The human AQP4 extracellular loop peptide (residues 135-153: LVTPPSVVGGLGVTMVHGN) was synthesized (ChinaPeptides, China) and stored lyophilized at −80 °C. An irrelevant control peptide (scrambled sequence of identical amino acid composition) was synthesized in parallel. For immunization, 1 mg peptide was dissolved in 20 μL dimethyl sulfoxide and then diluted to 1 mL with sterile Dulbecco’s phosphate-buffered saline (D-PBS). This solution was emulsified 1:1 (v/v) with complete Freund’s adjuvant containing 4 mg/mL heat-killed Mycobacterium tuberculosis H37Ra (Difco, USA) in incomplete Freund’s adjuvant (Sigma-Aldrich, USA). Female AQP4-KO mice (6–8 weeks) received two subcutaneous injections (50 μL per site) bilaterally at flank and shoulder regions (total 200 μL emulsion). 11 days post-immunization, inguinal, brachial, axillary, and cervical lymph nodes were harvested aseptically. Single-cell suspensions were prepared by mechanical dissociation through a 70-μm cell strainer using a syringe plunger, followed by red blood cell lysis and washing in RPMI 1640 medium supplemented with 10% fetal bovine serum, 1 mM sodium pyruvate, 1× non-essential amino acids, 10 mM HEPES and 50 μM β-mercaptoethanol. Cells were counted, resuspended at 2 × 10^6^ cells/mL, and seeded into 6-cm tissue culture dishes.

For Tfh differentiation, cells were stimulated with 10 μg/mL AQP4 peptide, 20 ng/mL IL-6, 20 ng/mL IL-21 (PeproTech, USA), 10 μg/mL anti-interferon-γ (IFN-γ) monoclonal antibody (mAb), 10 μg/mL anti-IL-4 mAb, and 20 μg/mL anti-TGF-β mAb (BioXcell, USA) (10, 11). For Th17 differentiation, cells were stimulated with 10 μg/mL AQP4 peptide, 5 ng/mL TGF-β, 10 ng/mL IL-1β, 10 ng/mL IL-6, 20 ng/mL IL-23 (PeproTech, USA), 10 μg/mL anti-IFN-γ mAb, and 10 μg/mL anti-IL-4 mAb (12). All cultures were maintained at 37°C in 5% CO_2_ for 72 h, resulting in approximately 10% polarization efficiency toward Tfh and Th17 cell lineages among viable cells. Differentiated cells were harvested, washed twice with sterile D-PBS, and resuspended in D-PBS for adoptive transfer. Recipient wild-type mice received 2 × 10^7^ viable cells via tail-vein injection. Control groups included (i) mice receiving an equivalent number of CD19^+^ B cells isolated from donor spleens (sorted by magnetic-activated cell sorting), (ii) mice injected with sterile D-PBS alone, (iii) donor mice immunized with irrelevant control peptide. All recipients received intraperitoneal injections of 200 ng pertussis toxin (Sigma-Aldrich) on day 0 and day 2 post-transfer to enhance blood–brain barrier permeability. Clinical disease onset and progression were monitored daily starting on day 5 post-transfer by a blinded observer using a standardized 5-point neurological disability scale: 0, no deficit; 0.5, partial loss of tail tone or slightly abnormal gait; 1.0, complete tail paralysis or both partial loss of tail tone and mild hind limb weakness; 1.5, complete tail paralysis and mild hind limb weakness; 2.0: tail paralysis with moderate hind limb weakness; 2.5, no weight-bearing on hind limbs but with some leg movement; 3.0, complete hind limb paralysis with no residual movement; 3.5, hind limb paralysis with mild weakness in forelimbs; 4.0, complete quadriplegia but with some movement of head; 4.5, moribund; 5.0, dead (13). Mice were euthanized at peak disease (day 10 post-transfer) by CO_2_ asphyxiation followed by cervical dislocation, and tissues were rapidly harvested for histopathological and immunological analyses. Sample sizes were determined based on previous similar studies and preliminary data (8, 14); ensuring N = 5 per group provides sufficient power to detect significant differences while minimizing animal use. Experiments were independently repeated twice, yielding similar results.

### 2.3 Flow cytometric identification of immune cell subsets

For Tfh cell identification, surface staining was performed using Zombie Aqua™ Fixable Viability Kit, anti-CD3-FITC, anti-CD4-BV421, anti-CD45-APC-Cy7, anti-CD11b-APC, anti-CXCR5-PE-Cy7, anti-ICOS-PE, and anti-PD1-BV605 (all from BioLegend, USA). Th17 cells were identified following 5h *in vitro* restimulation with Cell Stimulation Cocktail (eBioscience, USA) containing phorbol 12-myristate 13-acetate, ionomycin, and protein transport inhibitors. Surface markers were stained with Fixable Viability Dye eFluor™ 780 (eBioscience) and anti-CD3-FITC/anti-CD4-BV421; intracellular cytokines were detected after fixation/permeabilization (Foxp3/Transcription Factor Staining Buffer Set, eBioscience) using anti-IFN-γ-PE-Cy7, anti-IL-4-PE, and anti-IL-17A-APC (BioLegend). B cells were identified as live CD19^+^cells using anti-CD19-PE (Tonbo Biosciences, USA). All flow cytometry data were acquired on an Attune Acoustic Focusing Cytometer (Thermo Fisher Scientific, USA). Isotype control staining was performed to ensure accurate gating strategies.

### 2.4 Tissue processing and histopathological analysis

Mice were deeply anesthetized with 4% isoflurane and transcardially perfused with ice-cold Dulbecco’s phosphate-buffered saline (D-PBS) followed by 4% paraformaldehyde in 0.1 M phosphate buffer (pH 7.4). Optic nerves, lumbar spinal cord segments, and whole brains were dissected, post-fixed in 4% PFA at 4 °C for 24 h. Following fixation and washing, tissue specimens underwent standard histological processing, including graded ethanol dehydration, xylene-mediated clearing, and paraffin infiltration prior to microtome sectioning. Serial sections of 4-6-μm thickness were mounted onto charged glass slides. Hematoxylin and eosin (H&E) staining was performed according to the manufacturer’s instructions for the commercial H&E staining kit (Solarbio, China). For myelin visualization, Luxol fast blue (LFB) staining was carried out as previously reported (15). Briefly, deparaffinized and rehydrated sections were incubated overnight at 37 °C in 0.1% LFB solution (prepared in 95% ethanol containing 0.5% glacial acetic acid; Solarbio). Subsequently, sections were differentiated in 95% ethanol to remove excess dye, rinsed thoroughly with distilled water, treated with 0.05% lithium carbonate solution for 30 s, and finally dehydrated through a graded ethanol series and cleared in xylene before mounting.

For immunohistochemical analysis of murine tissue sections, avidin–biotin complex staining was performed using the following primary antibodies: anti-GFAP (1:1,000; Abcam, UK), anti-AQP4 (1:1,000; Sigma-Aldrich, USA), anti-myelin basic protein (MBP, 1:1,000; Abcam), anti-ionized calcium-binding adapter molecule 1 (IBA1, 1:1,000; Wako Chemicals, Japan), anti-CD3 (1:1,000; Abcam), anti-B220 (1:1,000; Abcam), anti-LY6G (1:1,000; Abcam), and anti-CXCL9 (1:1,000; Invitrogen, USA). Immunoreactive signals were visualized using standard ABC detection, and bright-field images were acquired using an inverted microscope (Olympus IX73, Japan).

For immunofluorescence staining, sections were incubated with primary antibodies against GFAP (1:1,000; Abcam) and CXCL9 (1:1,000; Invitrogen), followed by species-matched fluorescently labeled secondary antibodies. Nuclear counterstaining was performed with 4',6-diamidino-2-phenylindole (DAPI; Sigma-Aldrich, USA). Fluorescence images were acquired using a laser scanning confocal microscope (Olympus FV1200, Japan).

### 2.5 Flow cytometric analysis of central nervous system-infiltrating immune cells

At the peak of disease severity, mice were euthanized and brains and spinal cords were harvested for isolation of CNS-infiltrating immune cells. Cell suspensions were prepared and subjected to 70%-30% Percoll density gradient centrifugation, as previously described (16). Subsequent surface immunostaining was performed using the following fluorochrome-conjugated antibodies: Zombie Aqua™ Viability Dye (to discriminate live from dead cells), anti-CD3-FITC, anti-CD4-BV421, anti-CD45-APC-Cy7, anti-CD11b-APC, anti-CXCR5-PE-Cy7, anti-PD1-BV605, and anti-CD19-PE (BioLegend, USA). Stained cells were acquired on an Attune Acoustic Focusing Cytometer (Thermo Fisher Scientific, USA). Isotype control staining was performed to ensure accurate gating strategies.

### 2.6 RNA library construction and transcriptome sequencing

Total RNA was extracted from biological replicates of the lumbar spinal cord enlargement (N = 5 per group) and from primary astrocytes treated with either purified human AQP4-ab or PBS control (N = 3 per group), using TRIzol^®^ Reagent (Invitrogen, USA). The RNA concentration was measured with a K5500 spectrophotometer, and its integrity was assessed using an Agilent 2200 TapeStation system. Poly(A)+ mRNA was enriched using oligo(dT) magnetic beads, followed by fragmentation to an average length of ~200 nucleotides. First- and second-strand cDNA synthesis was performed, and sequencing adapters were ligated prior to low-cycle PCR amplification. Libraries were constructed using the Nextera XT DNA Library Preparation Kit (Illumina, USA) and sequenced on an Illumina HiSeq 2500 platform (paired-end, 150 bp) (RiboBio, China). The RNA-seq data presented were from one representative experiment.

### 2.7 Bioinformatics analysis

Differential gene expression analysis was conducted using DESeq2 (R package version 1.46.0) (17). Genes exhibiting |log_2_(fold change)| > 1 and an adjusted P-value (Q-value) < 0.05 were designated differentially expressed genes (DEGs) and ranked by descending |log_2_(fold change)|. Functional enrichment analyses—including Gene Ontology (GO) terms across biological process (BP), molecular function (MF), and cellular component (CC) domains—and Kyoto Encyclopedia of Genes and Genomes (KEGG) pathway enrichment were performed using clusterProfiler (R package version 4.14.6) (18, 19). Gene set enrichment analysis (GSEA) was additionally applied against the KEGG pathway database.

### 2.8 Quantitative real-time PCR

Total RNA was isolated from lumbar spinal cord enlargements using TRIzol^®^ Reagent (Invitrogen, USA). Complementary DNA was synthesized from 1 µg of total RNA using the PrimeScript™ RT reagent Kit (TaKaRa Bio, Japan). Quantitative real-time PCR (qRT-PCR) was performed on a Roche LightCycler 480 System using SYBR^®^ Premix Ex Taq™ II (TaKaRa Bio, Japan). Amplification specificity was confirmed by melting curve analysis. GAPDH served as the endogenous reference gene, and relative mRNA expression levels were calculated using the 2^−ΔΔCT^ method. All primer sequences were synthesized (BioTNT, China) and listed below:

GAPDH sense: 5'-GACACTGAGCAAGAGAGGCCCTA-3', antisense: 5'-TGGGATGGAAATTGTGAGGGA-3'; CXCL9 sense: 5'-GCAGAAGTTCCGTCTTGAGCA-3', antisense: 5'-CCAGCAGCACAAAAACCACC-3'; CXCR3 sense: 5'-AAGTGCTAGATGCCTCGGACT-3', antisense: 5'-GGGAGTCAGAGAAGTCGCTCT-3'; BCL-6 sense: 5'-TACCCAAAGGATGCTGTAACAC-3'; antisense: BCL-6 downstream: 5'-CCCCACCCCAACTATGATT-3'; PRDM1 sense: 5'-CAAGTTCCTGTTGCCACCGT-3', antisense: 5'-TGCTGCCACTAAGGAGGTTACTG-3'; T-bet sense: 5'-AGTGATTGGTTGGAGAGGAAG-3'; antisense: 5'-GGGACACTCCTGTCGTATTTC-3'; GATA3 sense: 5'-GAGAGACTGAGAGAGCGAGAC-3'; antisense: 5'-TGAGTAGCAAGGAGCGTAGA-3'; RORC sense: 5'-GC CTTCCCTCCACTCTATAAG-3'; RORC antisense: 5'-AGAAAGTTGTCCTTCCTCCAG-3'.

### 2.9 Statistical analysis

Flow cytometry data were analyzed using FlowJo X10.0 software (FlowJo, USA) to determine cell population frequencies and absolute counts. Immunohistochemical images were quantified using ImageJ software (National Institutes of Health, USA) to calculate mean optical density (OD) values. qRT-PCR cycle threshold (Ct) values were extracted using QuantStudio Design and Analysis Software (Thermo Fisher Scientific, USA). Quantitative colocalization analysis was performed using ImageJ with the Coloc2 plugin on confocal images double-labeled for CXCL9 and GFAP. We analyzed five randomly selected fields of view. Pearson’s correlation and Manders’ coefficients were calculated to assess the degree of colocalization between CXCL9 and GFAP signals. Statistical analysis was performed in SPSS 22.0 (SPSS Inc., USA) and R (version 4.1.3, http://www.r-project.org/), and graphs were generated using GraphPad Prism 7 software (GraphPad Software Inc., USA) and R. Data were presented as mean ± standard deviation (SD). Group comparisons were conducted using one-way ANOVA (with homogeneity of variance verified by Levene’s test) or Welch’s ANOVA when variances were unequal. *Post-hoc* pairwise comparisons were performed using Tukey’s honestly significant difference test or the Games-Howell test, respectively. A two-tailed P-value of < 0.05 was considered statistically significant.

## 3 Results

### 3.1 Adoptive transfer of Tfh and Th17 cells induces NMOSD-like disease in recipient mice

**Figure 1:**
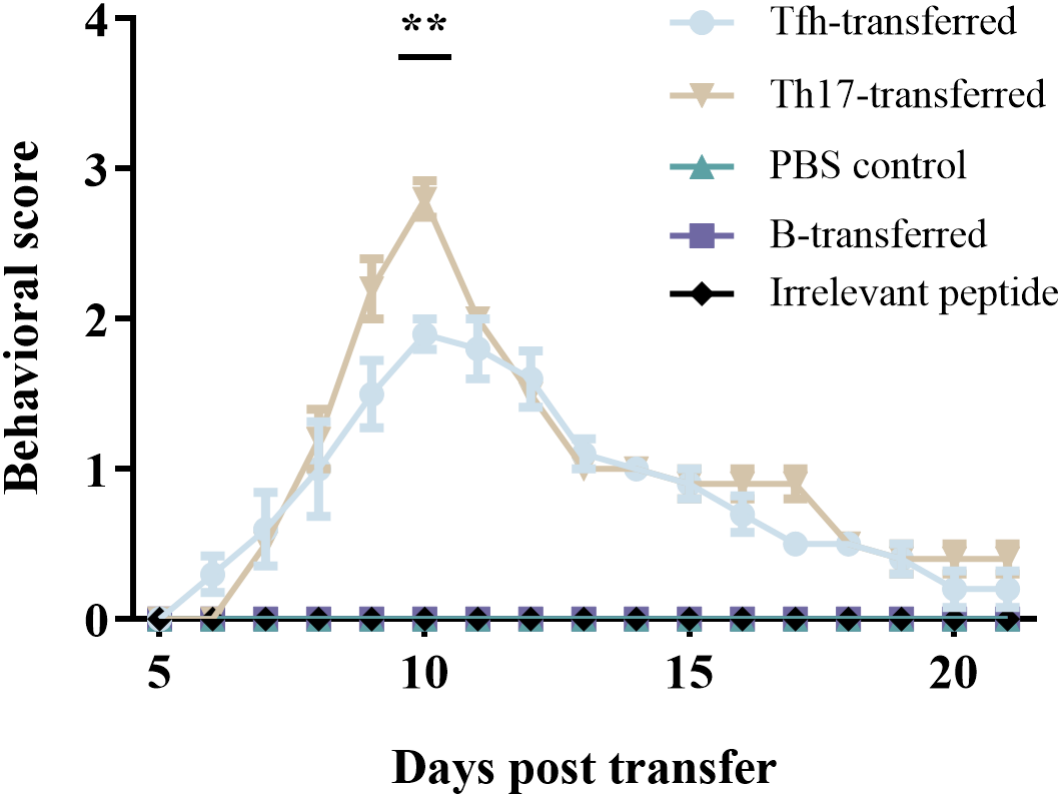
Clinical scores of mice from Tfh-transferred, Th17-transferred, and three control groups from days 5 to 21 post-transfer (N = 5 per group). Data are presented as mean ± SEM. *P < 0.05, **P < 0.01, ***P < 0.001.

We first polarized primary cells isolated from mouse lymph nodes under defined cytokine conditions over 3 days to generate highly enriched Tfh and Th17 cell populations. Gating strategies for the identification of Tfh, Th17, and B cells are comprehensively described in **Supplementary Figures 1**-**3**. Following intravenous adoptive transfer into syngeneic recipient mice, both Tfh and Th17 cell populations induced a reproducible NMOSD-like phenotype. Clinical manifestations emerged as early as day 6 post-transfer, beginning with mild tail paresis that progressively worsened. By day 10, disease severity peaked, characterized by pronounced flaccid paralysis of the tail and hind limbs or even complete paraplegia. Mean clinical scores at peak disease were 1.9 ± 0.2 in the Tfh-transferred group and 2.8 ± 0.2 in the Th17-transferred group, with a statistically significant difference between groups (Th17 vs. Tfh: P = 0.003, **Movie S1 and S2**). Thereafter, symptoms gradually resolved: by day 21, clinical scores declined significantly to 0.2 ± 0.2 (Tfh) and 0.4 ± 0.2 (Th17) (**Figure 1**). In contrast, control mice exhibited no neurological deficits throughout the 21-day observation period, confirming that pathogenicity is specifically attributable to AQP4-reactive Tfh and Th17 cells.

### 3.2 Tfh and Th17 cell transfer drives region-specific neuroinflammation and demyelination

**Figure 2:**
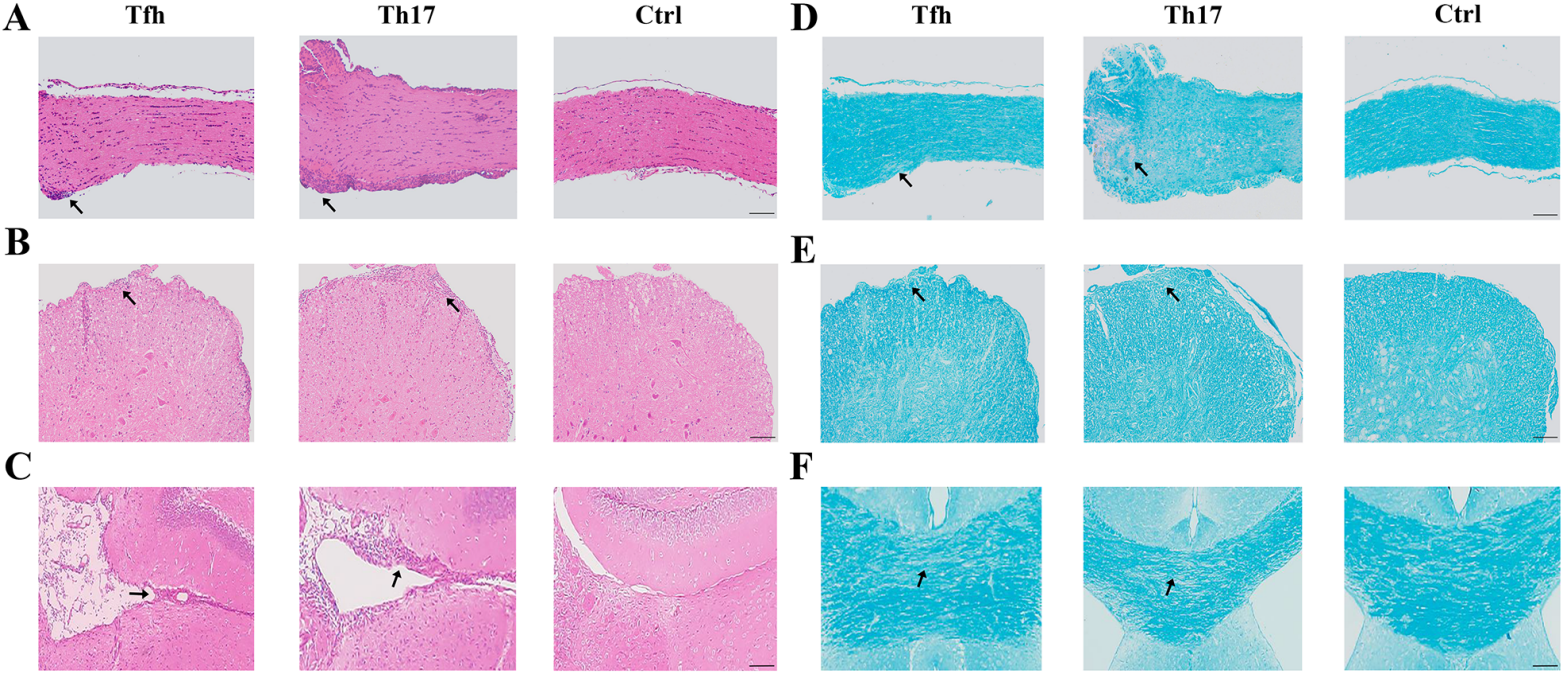
Histological staining of the optic nerve, spinal cord, and brain from Tfh- transferred, Th17-transferred, and PBS control groups. **(A–C)**. HE staining of the optic nerve **(A)**, spinal cord **(B)**, and brain third ventricle **(C–F)**. LFB staining of the optic nerve **(D)**, spinal cord **(E)**, and brain corpus callosum **(F)**. Scale bars = 100 μm.

**Figure 3:**
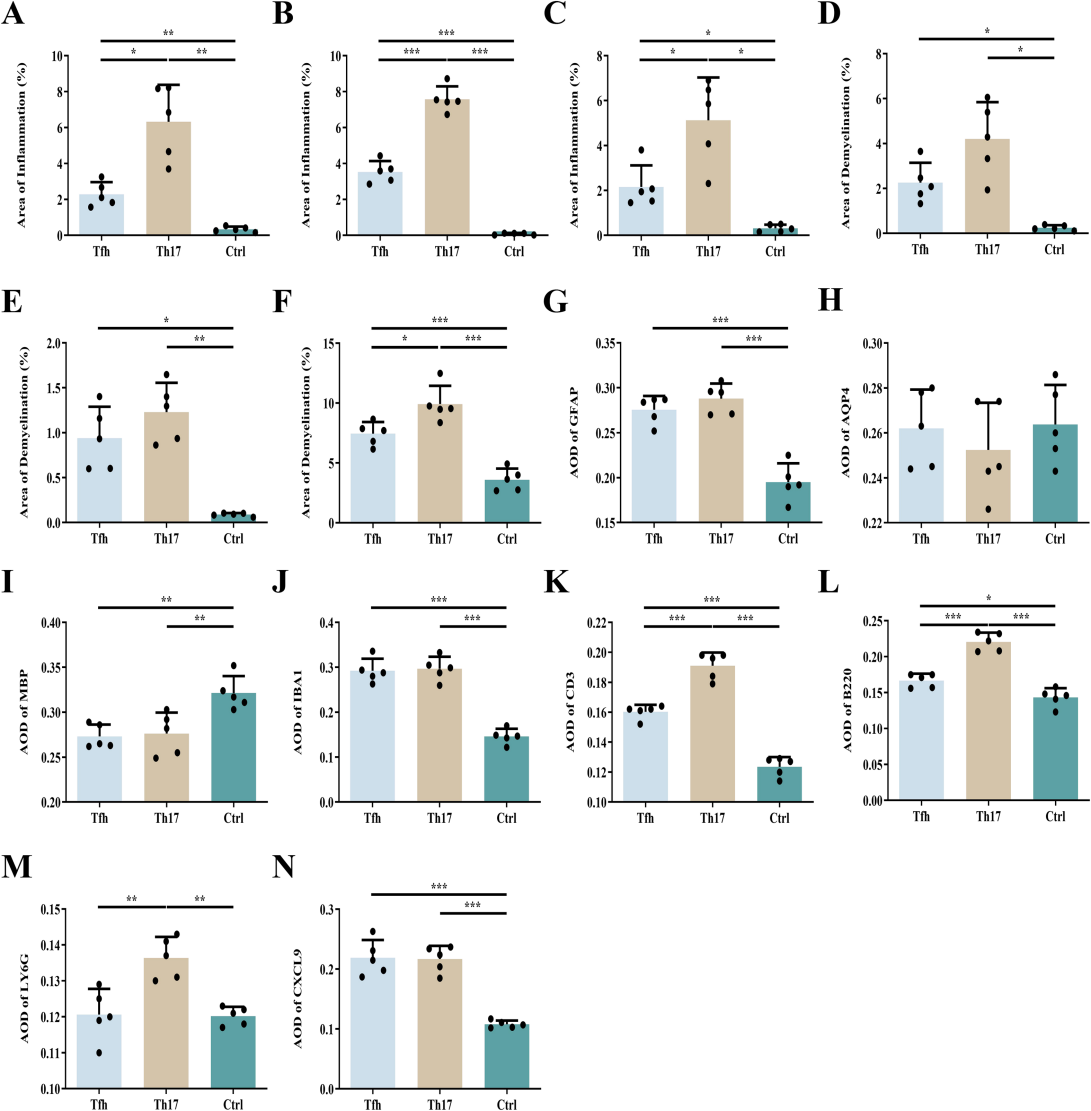
Comparisons of the inflammation and demyelination in the optic nerve, spinal cord and brain and AOD of various antigens in the spinal cord from Tfh-, Th17-transferred and PBS control groups (N = 5 per group). **(A)** Area of inflammation in the optic nerve; **(B)** Area of inflammation in the spinal cord; **(C)** Area of inflammation in the brain; **(D)** Area of demyelination in the optic nerve; E. Area of demyelination in the spinal cord; **(F)** Area of demyelination in the brain; **(G)** AOD of GFAP; **(H)** AOD of AQP4; **(I)** AOD of MBP; **(J)** AOD of IBA1; **(K)** AOD of CD3; **(L)** AOD of B220; **(M)** AOD of LY6G; **(N)** AOD of CXCL9. *P < 0.05, **P < 0.01, ***P < 0.001.

Histopathological assessment of optic nerve, spinal cord, and brain tissues revealed robust inflammatory infiltration and demyelination in both Tfh- and Th17-transferred mice relative to PBS controls. H&E staining demonstrated dense perivascular and parenchymal immune cell accumulation in the optic chiasm, spinal cord white matter (particularly anterior and lateral funiculi), and brain third ventricle (**Figures 2A–C**). Quantitative analysis confirmed statistically significant increases in inflammatory burden across all regions: optic nerve (Tfh vs. Ctrl: P = 0.005; Th17 vs. Ctrl: P = 0.006), spinal cord (both comparisons: P < 0.001), and brain (Tfh vs. Ctrl: P = 0.026; Th17 vs. Ctrl: P = 0.010) (**Figures 3A–C**). Notably, spinal cord and brain inflammations were significantly more severe in Th17-transferred mice than in Tfh-transferred mice (optic nerve: P = 0.021; spinal cord: P < 0.001; brain: P = 0.049). LFB staining further revealed substantial demyelination localized to the optic nerve margins, spinal cord peripheral white matter, and brain corpus callosum (**Figures 2D–F**). Demyelination was significantly elevated versus PBS controls in all regions: optic nerve (Tfh vs. Ctrl: P = 0.015; Th17 vs. Ctrl: P = 0.012), spinal cord (Tfh vs. Ctrl: P = 0.012; Th17 vs. Ctrl: P = 0.003), and brain (Tfh vs. Ctrl: P < 0.002; Th17 vs. Ctrl: P < 0.001) (**Figures 3D–F**). Collectively, these data demonstrate that AQP4-reactive Tfh and Th17 cells drive CNS-targeted neuroinflammation and demyelination, with Th17 cells exhibiting heightened tropism for spinal cord and brain pathology.

### 3.3 Tfh and Th17 cell transfer activates glial cells and promotes immune cell infiltration in the spinal cord

**Figure 4:**
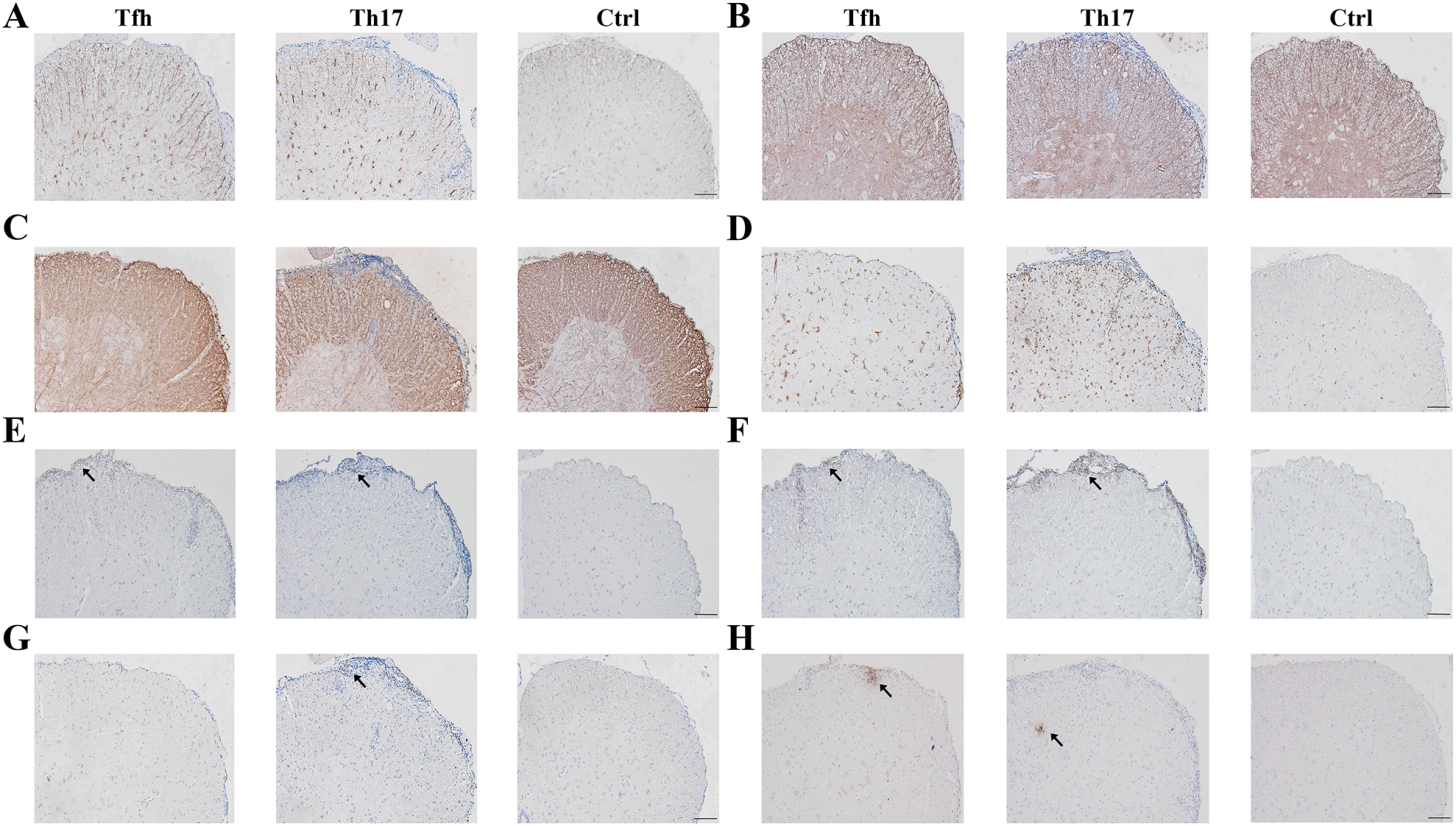
Immunohistochemistry of various antigens in the spinal cord from Tfh- transferred, Th17-transferred, and PBS control groups. **(A)** GFAP staining; **(B)** AQP4 staining; **(C)** MBP staining; **(D)** IBA1 staining; **(E)** CD3 staining; **(F)** B220 staining; **(G)** LY6G staining; **(H)** CXCL9 staining. Scale bars = 100 μm.

Immunohistochemical analysis of spinal cord sections showed marked upregulation of glial activation markers and immune cell signatures in both Tfh- and Th17-transferred groups relative to PBS controls (**Figures 4A–H**). Compared with PBS controls, both experimental groups exhibited significantly higher average optical density (AOD) for GFAP (astrocyte activation; P < 0.001 for both), IBA1 (microglial activation; P < 0.001 for both), CD3 (T-cell infiltration; P < 0.001 for both), B220 (B-cell infiltration; Tfh vs. Ctrl: P = 0.021; Th17 vs. Ctrl: P < 0.001), and CXCL9 (pro-inflammatory chemokine; P < 0.001 for both). Conversely, MBP expression—indicative of myelin integrity—was significantly reduced (Tfh vs. Ctrl: P = 0.004; Th17 vs. Ctrl: P = 0.007), corroborating demyelination. Importantly, Th17-transferred mice displayed significantly greater AOD for CD3 (P < 0.001), B220 (P < 0.001), and LY6G (neutrophil marker; P = 0.002) than Tfh-transferred mice (**Figures 3G–N**), suggesting enhanced recruitment of adaptive and innate immune effectors.

### 3.4 Tfh and Th17 cell transfer enriches distinct CNS-infiltrating immune subsets

**Figure 5:**
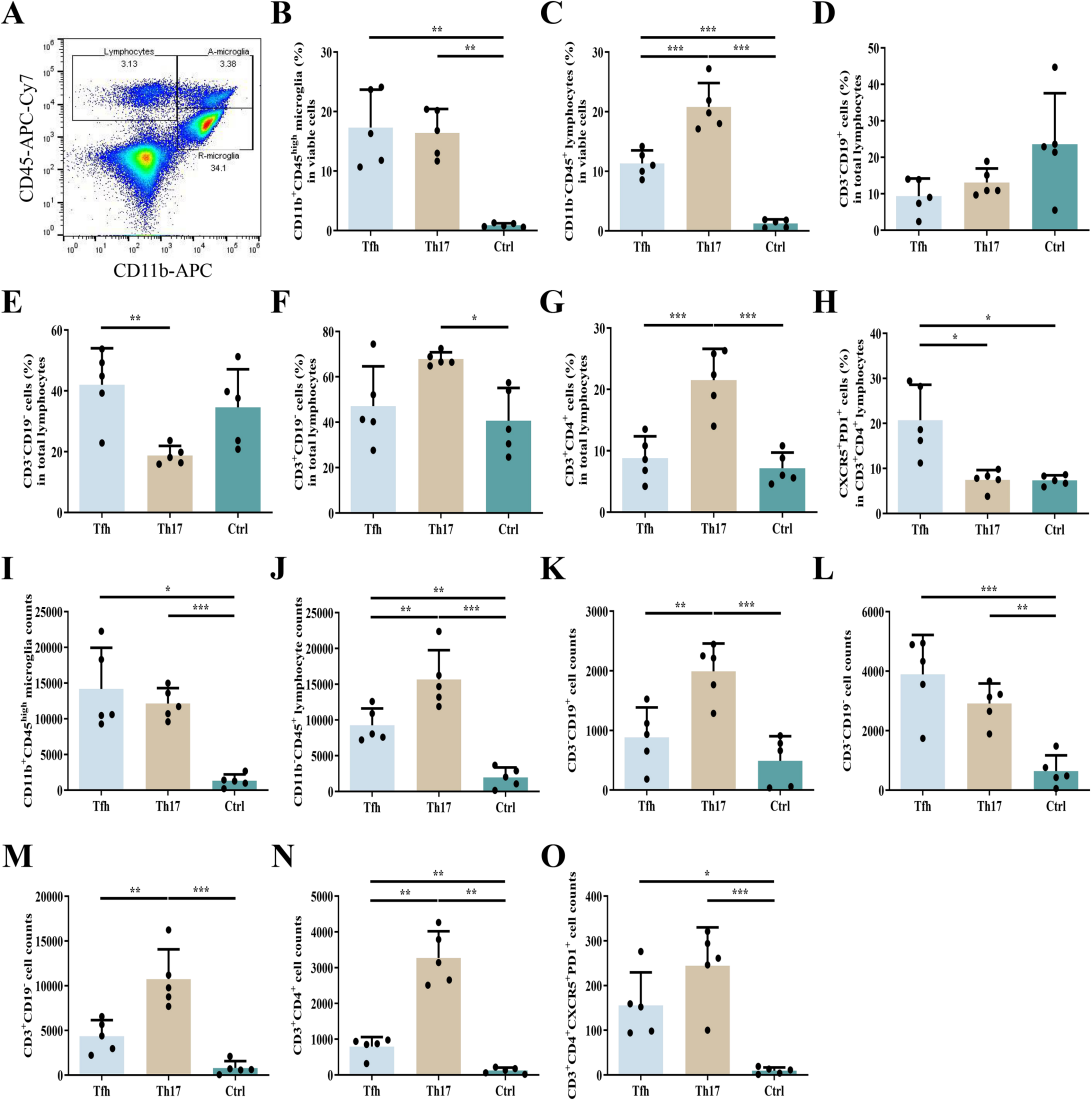
Comparisons of proportions and counts of infiltrating lymphocyte subsets and microglia/macrophage in the central nervous system from Tfh- transferred, Th17-transferred and PBS control groups (N = 5 per group). **(A)** Gating the CD11b^+^CD45^high^ microglia/macrophage, CD11b^+^CD45^low^ microglia/macrophage, and CD11b^−^CD45^+^ lymphocytes; **(B)** Proportion of CD11b^+^CD45^high^ microglia/macrophage; **(C)** Proportion of CD11b^−^CD45^+^ lymphocytes; **(D)** Proportion of CD3^−^CD19^+^ cells; **(E)** Proportion of CD3^−^CD19^−^ cells; **(F)** Proportion of CD3^+^CD19^−^ cells; **(G)** Proportion of CD3^+^CD4^+^ cells; **(H)** Proportion of CXCR5^+^PD1^+^ cells. **(I)** CD11b^+^CD45^high^ microglia/macrophage counts; **(J)** CD11b^−^CD45^+^ lymphocyte counts; **(K)** CD3^−^CD19^+^ cell counts; **(L)** CD3^−^CD19^−^ cell counts; **(M)** CD3^+^CD19^−^ cell counts; **(N)** CD3^+^CD4^+^ cell counts; **(O)** CD3^+^CD4^+^CXCR5^+^PD1^+^ cell counts. *P < 0.05, **P < 0.01, ***P < 0.001.

Flow cytometric profiling of CNS-infiltrating leukocytes revealed substantial expansion of multiple immune populations in both Tfh- and Th17-transferred mice (**Supplementary Figure 4**). Relative to PBS controls, both groups exhibited significantly increased frequencies of CD11b^+^CD45^high^ microglia/macrophages (Tfh vs. Ctrl: P = 0.010; Th17 vs. Ctrl: P = 0.002) and CD11b^−^CD45^+^ lymphocytes (both: P < 0.001). Furthermore, the Th17 group showed significantly higher proportions of CD11b^−^CD45^+^ lymphocytes (P < 0.001) and CD3^+^CD4^+^ T cells (P < 0.001) compared with the Tfh group. In addition, CXCR5^+^PD1^+^ cells—a phenotypic signature associated with Tfh-like or germinal center-experienced T cells—were significantly enriched in the Tfh group (Tfh vs. Th17: P = 0.038; Tfh vs. Ctrl: P = 0.040) (**Figures 5B–H**).

Quantitative analysis revealed a significant increase in the absolute counts of CD11b^+^CD45^high^ microglia/macrophages (P = 0.015 for Tfh vs. Ctrl; P < 0.001 for Th17 vs. Ctrl), CD11b^−^CD45^+^ lymphocytes (P = 0.004 for Tfh vs. Ctrl; P < 0.001 for Th17 vs. Ctrl), CD3^−^CD19^+^ cells (P < 0.001 for Th17 vs. Ctrl), CD3^+^CD4^+^ cells (P = 0.008 for Tfh vs. Ctrl; P = 0.001 for Th17 vs. Ctrl), and CD3^+^CD4^+^CXCR5^+^PD1^+^ cells (P = 0.011 for Tfh vs. Ctrl; P < 0.001 for Th17 vs. Ctrl). Furthermore, the Th17 group exhibited significantly higher absolute counts than the Tfh group for CD11b^−^CD45^+^ lymphocytes (P = 0.010), CD3^−^CD19^+^ cells (P = 0.007), CD3^+^CD19^−^ cells (P = 0.002), and CD3^+^CD4^+^ cells (P = 0.002) (**Figures 5I–O**). These findings indicate that while both Tfh and Th17 cells promote broad CNS immune infiltration, Th17 cells elicit a quantitatively and phenotypically distinct inflammatory milieu, particularly enriched in conventional T helper subsets and B cell-interacting T cells.

### 3.5 Transcriptome sequencing identifies CXCL9 as a shared differentially expressed gene

**Figure 6:**
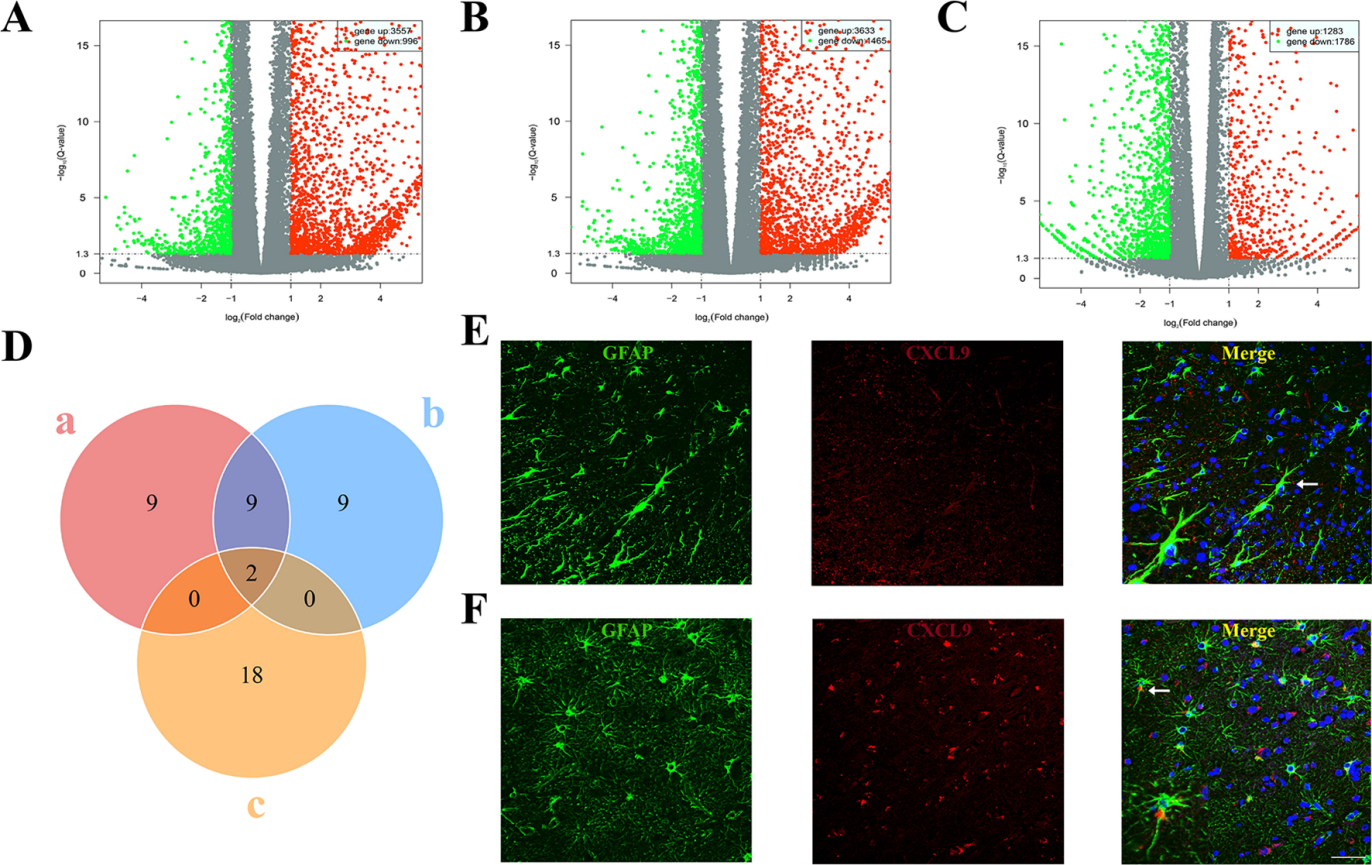
Venn diagram of top 20 DEGs and confocal imaging of GFAP and CXCL9 in the mouse spinal cord and human brain. **(A)** Volcano plot of DEGs from the comparison between Tfh-transferred and PBS control groups. **(B)** Volcano plot of DEGs from the comparison between Th17-transferred and PBS control groups. **(C)** Volcano plot of DEGs from the comparison between primary astrocytes treated with AQP4-ab and PBS control. **(D)** Top 20 DEGs were screened from the comparisons between Tfh-transferred and PBS control groups (a, N = 5 biological replicates per group), Th17-transferred and PBS control groups (b, N = 5 biological replicates per group), and primary astrocytes treated with AQP4-ab and PBS control (c, N = 3 biological replicates per group). Experiments were repeated independently with similar results. The only two overlapping genes were *Cxcl9* and *Saa3*. **(E)** Confocal imaging of GFAP (green) and CXCL9 (red) in the mouse spinal cord. **(F)** Confocal imaging of GFAP (green) and CXCL9 (red) in the human brain. Scale bars = 100 μm.

RNA sequencing was performed on biological replicates of lumbar spinal cord enlargements and primary astrocytes to identify DEGs, with 3,557, 3,663, and 1,283 upregulated genes and 996, 1,465, and 1,786 downregulated genes from the comparisons between Tfh-transferred and PBS control groups, Th17-transferred and PBS control groups, and primary astrocytes treated with AQP4-ab and PBS control, respectively (**Figures 6A–C**). Top 20 DEGs were compiled in **Table 1** and visualized using Venn diagrams (**Figure 6D**). Only two genes were consistently upregulated across all comparisons: *Cxcl9* and *Saa3*. A comprehensive list of all DEGs, along with enriched BPs, CCs, and MFs derived from GO enrichment analyses, is provided in **Supplementary Tables 1**-**S12**. The top significantly enriched BPs, CCs, and MFs identified in the comparison between Tfh-transferred and PBS control groups were positive regulation of cytokine production, membrane raft, and immune receptor activity, respectively. In the comparison between Th17-transferred and PBS control groups, the top enriched terms were positive regulation of response to external stimulus, receptor complex, and immune receptor activity. In primary astrocytes treated with AQP4-ab and PBS control, the top enriched terms were positive regulation of defense response, extracellular matrix, and receptor ligand activity. KEGG pathway enrichment analyses—including both conventional DEG-based and GSEA-based approaches—are summarized in **Supplementary Tables 13**-**S18**. Comparative pathway enrichment analyses revealed that the cytokine–cytokine receptor interaction pathway was consistently among the top significantly enriched KEGG pathways across all experimental comparisons. Specifically, in the Tfh-transferred versus control group comparison, this pathway ranked first both in conventional KEGG enrichment analysis and in GSEA. In the Th17-transferred versus control group comparison, coronavirus disease-COVID-19 was the top enriched pathway in KEGG analysis, whereas cytokine–cytokine receptor interaction emerged as the top enriched pathway in GSEA. Similarly, in primary astrocytes treated with AQP4-ab versus PBS-treated controls, cytokine–cytokine receptor interaction was the top enriched pathway in KEGG analysis, whereas the TNF signaling pathway ranked highest in GSEA. The above results identified which transcriptional changes are common versus distinct between T-cell-mediated and antibody-mediated astrocyte responses. The comparisons helped dissect the relative contributions of each pathway in NMOSD pathogenesis.

**Table 1:** Top 20 differentially expressed genes from the comparisons between Tfh-transferred and PBS control groups, Th17-transferred and PBS control groups, and primary astrocytes treated with AQP4-ab and PBS control.

Groups	Tfh-Ctrl	Th17-Ctrl	AQP4-Ctrl
1	Sirpb1c	Cxcl13	Cxcl3
2	Alb	Icos	Saa3
3	Gpr141	Sirpb1c	Lcn2
4	Cd200r4	Saa3	Cxcl9
5	Icos	Cd8b1	Il1a
6	Ifi205	Cd200r4	Cxcl1
7	Il1rn	Trbc1	Ccl5
8	Arg1	Alb	Il12b
9	Saa3	Izumo1r	Ms4a4d
10	Ly6i	Arg1	Nos2
11	Ifng	Ly6i	Cxcl11
12	Dpep2	Clec4d	Gpr18
13	Cxcl9	Dpep2	Il6
14	Slamf7	Cxcl9	Gm14275
15	Gm9733	Tgm1	Gm31522
16	Trem1	Klrb1b	Pglyrp2
17	Slfn1	Gm4841	Cxcl2
18	Serpina3f	Gbp2b	Pilrb2
19	Acod1	Gzmk	Mmp13
20	Gbp2b	Gpr141	Csf3

Top 20 differentially expressed genes are selected based on the absolute value of Log2FC after filtering for significance (Q-value < 0.05).

### 3.6 Confocal colocalization analysis confirms the astrocytic origin of CXCL9

Quantitative colocalization analysis revealed a positive correlation between CXCL9 and GFAP signals in the mouse spinal cord [Pearson’s coefficient = 0.20 ± 0.02, Manders’ coefficients (M1) = 0.91 ± 0.01] (**Figures 6B–D**) and human brain tissue [Pearson’s coefficient = 0.22 ± 0.03, Manders’ coefficients (M1) = 0.90 ± 0.02] (**Figures 6E–G**), supporting astrocyte-specific production and secretion of CXCL9.

### 3.7 Adoptive transfer of Tfh and Th17 cells enhances chemotactic signaling and promotes B-cell differentiation

**Figure 7:**
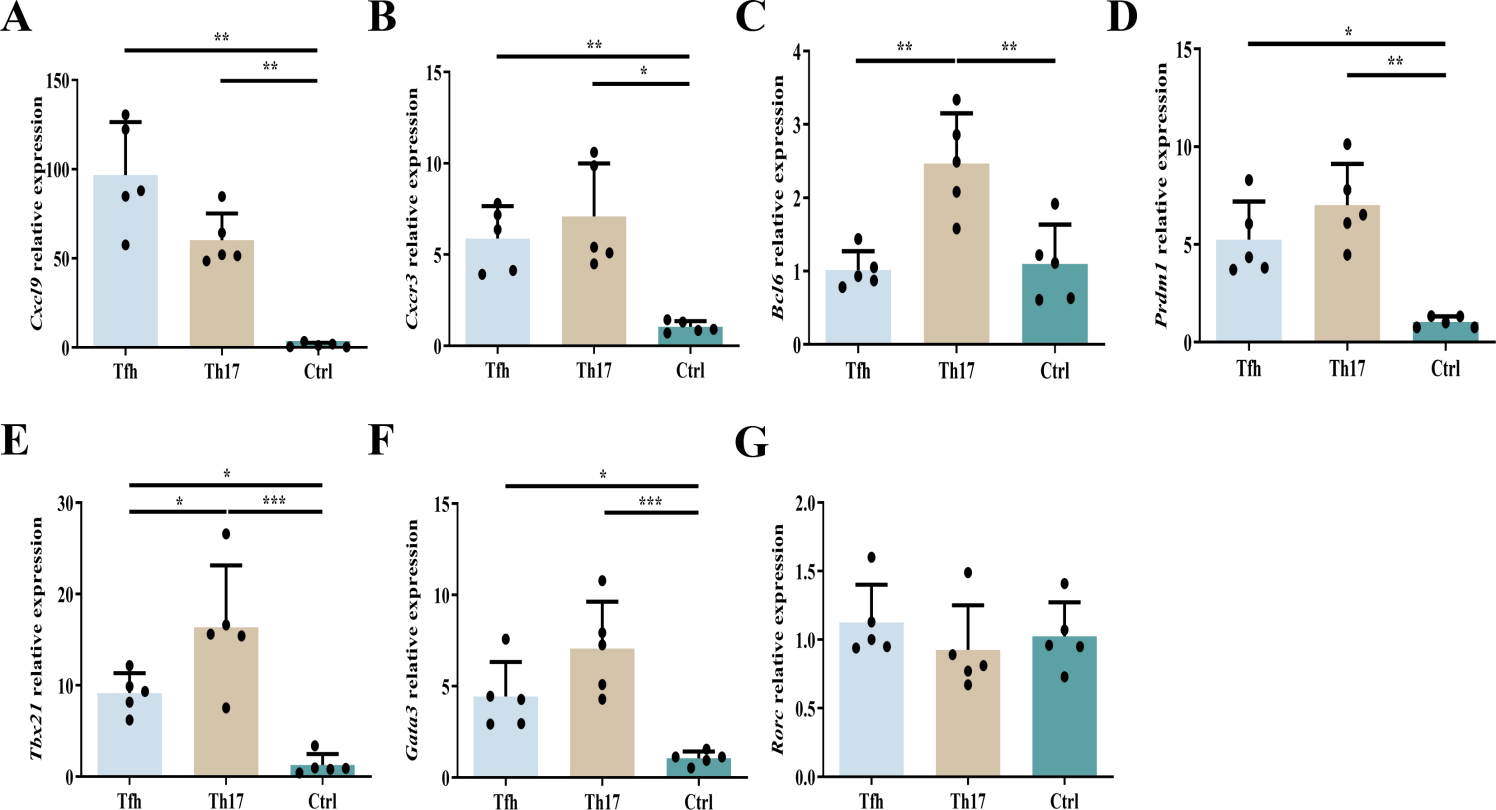
Comparison of relative expression of various RNA in the spinal cord from Tfh-transferred, Th17-transferred, and PBS control groups (N = 5 per group). **(A)** Relative expression of *Cxcl9*. **(B)** Relative expression of *Cxcr3*. **(C)** Relative expression of *Bcl6*. D. Relative expression of *Prdm1*. **(E)** Relative expression of *Tbx21*. **(F)** Relative expression of *Gata3*. **(G)** Relative expression of *Rorc*. *P < 0.05, **P < 0.01, ***P < 0.001.

qRT-PCR demonstrated significantly elevated mRNA expression of key chemotaxis- and B-cell differentiation-associated genes in both Tfh and Th17 groups relative to PBS controls. Specifically, *Cxcl9* (P = 0.004 for Tfh vs. Ctrl; P = 0.002 for Th17 vs. Ctrl), *Cxcr3* (P = 0.007 for Tfh vs. Ctrl; P = 0.020 for Th17 vs. Ctrl), *Prdm1* (P = 0.017 for Tfh vs. Ctrl; P = 0.007 for Th17 vs. Ctrl), *Tbx21* (encoding T-bet; P = 0.029 for Tfh vs. Ctrl; P < 0.001 for Th17 vs. Ctrl), and *Gata3* (P = 0.034 for Tfh vs. Ctrl; P < 0.001 for Th17 vs. Ctrl) were all significantly upregulated. Moreover, the Th17 group showed significantly higher expression of *Bcl6* (P = 0.002) and *Tbx21* (P = 0.045) compared with the Tfh group, whereas *Rorc* expression remained unchanged (**Figures 7A–G**).

## 4 Discussion

This study successfully established murine models of NMOSD via adoptive transfer of antigen-specific Tfh and Th17 cells, respectively. Recipient mice developed progressive hind-limb weakness accompanied by inflammatory cell infiltration and demyelination in the spinal cord and optic nerve. Histopathological and clinical assessments revealed that disease severity—including motor deficits, inflammatory burden, and extent of demyelination—was significantly greater in the Th17-transferred group compared with the Tfh-transferred group. While the pathology observed (e.g., neuroinflammation and demyelination) shares features with NMOSD, it likely represents the inflammatory sequela of T-cell activation, which may act synergistically with, or parallel to, humoral factors in the actual human condition.

For immunization, we selected the human AQP4 peptide p135-153, corresponding to the extracellular C-loop domain—a region consistently identified as the dominant epitope targeted by pathogenic AQP4 autoantibodies in NMOSD patients (20). Supporting this choice, prior studies have delineated four major immunodominant AQP4 epitopes (p137-151, p222-236, p217-231, and p269-283) capable of eliciting T-cell responses in NMOSD; among these, p137–151 and p222–236 exhibit the highest functional sensitivity and prevalence in patient-derived T-cell assays (21). The p135–153 sequence overlaps substantially with p137–151 and preserves key conformational and linear determinants critical for T-cell recognition, thereby justifying its use in preclinical modeling.

To ensure robust generation of AQP4-reactive T cells, we employed AQP4-KO mice—not wild-type (WT) controls—as donors for T-cell isolation and *in vitro* priming. In WT mice, central and peripheral tolerance mechanisms effectively suppress the activation and expansion of high-affinity AQP4-reactive T cells; in contrast, AQP4-KO mice lack thymic deletion of AQP4-specific T-cell clones, enabling their survival, expansion, and functional differentiation upon antigen exposure (14). Furthermore, WT mice may harbor AQP4-specific regulatory T cells or other immunomodulatory mechanisms that actively constrain autoreactive T-cell responses—an effect absent in AQP4-KO donors (8).

Notably, disease induction in our model was achieved solely through adoptive transfer of AQP4-reactive Tfh or Th17 cells—without exogenous administration of AQP4-ab. This aligns with established evidence, indicating that while AQP4-ab potently exacerbates neuroinflammation in EAE-based models, it is insufficient to initiate disease *de novo* in immunocompetent mice (22). Rather, AQP4-ab acts downstream: once AQP4-reactive T cells breach the blood–central nervous system barrier and trigger local inflammation in the optic nerve and spinal cord, B-cell-derived AQP4-ab amplifies pathology via classical complement activation and neutrophil/macrophage recruitment (8).

Th17 cells are well-established mediators of blood–CNS barrier disruption and CNS inflammation in AQP4-ab-positive NMOSD (23). Consistent with prior reports, adoptive transfer of AQP4-specific Th17 cells recapitulated core NMOSD features—including acute paralysis, perivascular inflammation, and demyelination (8, 14). Similarly, recipients of AQP4-specific Tfh cells developed transient neurological deficits followed by spontaneous clinical remission over a 21-day observation period. However, the Th17 group exhibited significantly higher cumulative clinical scores, more pronounced inflammatory infiltration, and greater tissue damage than the Tfh group—despite comparable demyelination (8, 14). Additional evidence supports a pathogenic contribution of Tfh cells in NMOSD: one study demonstrated that AQP4 immunization via electroporation induced Tfh-dependent germinal center formation and autoantibody production, culminating in NMOSD-like pathology (24). Yet, in EAE models, Th17-driven disease consistently yields higher clinical scores than Tfh-driven disease—suggesting differential effector potency (11). Importantly, spontaneous recovery observed in WT recipients of AQP4-reactive T cells has been linked to activation-induced apoptosis of donor T cells, whereas myelin oligodendrocyte glycoprotein (MOG)-reactive T cells persist and sustain chronic disease—highlighting epitope- and context-dependent differences in T-cell longevity and pathogenicity (25). Collectively, these findings indicate that while AQP4-reactive Tfh cells primarily serve a helper role in supporting B-cell activation and autoantibody production, they also possess intrinsic, albeit milder, encephalitogenic capacity distinct from that of Th17 cells.

GFAP is a cytoskeletal intermediate filament protein predominantly expressed in astrocytes, where it contributes to structural integrity and cellular motility. Notably, GFAP expression is markedly upregulated in reactive astrocytes—consistent with previous reported findings (26, 27). Immunohistochemical analysis revealed significant astrocytic activation in both the Tfh and Th17 adoptive transfer groups. As key innate immune effectors of the CNS, astrocytes can sense proinflammatory cytokines secreted by CNS-infiltrating lymphocytes, subsequently producing chemokines that recruit peripheral immune cells into the CNS parenchyma. This cascade may perpetuate chronic neuroinflammation and contribute to progressive neurodegeneration (28, 29). In contrast to antibody-mediated pathology, AQP4 loss was not observed in either group; such loss typically requires the presence of AQP4-ab, rather than T-cell activity alone—a finding aligned with prior experimental and clinical evidence (8, 30). MBP, a major structural component of CNS myelin essential for myelin sheath compaction and stability (31), exhibited mild demyelination in both the Tfh and Th17 groups—indicating that AQP4-ab-independent mechanisms can drive early myelin injury. IBA1, a microglia- and macrophage-specific marker whose expression increases upon activation (32), showed robust upregulation in both groups, confirming pronounced microglial activation. This observation supports a pathogenic role for microglia in this model. Indeed, histopathological analyses of early-stage AQP4-ab-positive NMOSD patients have similarly demonstrated marked microglial activation in AQP4-rich CNS regions (33). Comparable microglial hyperactivation has also been documented in murine NMOSD models induced by intracerebroventricular or intraspinal injection of AQP4-ab (34). Cellular infiltration patterns differed between groups: the Tfh group exhibited modest infiltration by T and B lymphocytes with minimal neutrophil involvement, whereas the Th17 group displayed substantially greater lymphocytic infiltration—including both T and B cells—and prominent neutrophil recruitment (19, 35). These distinctions suggest divergent immunopathogenic mechanisms underlying the two models. Flow cytometric analyses corroborated these histological findings, revealing significantly increased proportions and absolute numbers of activated microglia/macrophages in both groups. Moreover, the Th17 group contained markedly higher frequencies and total counts of infiltrating lymphocytes—including T and B cells—compared with the Tfh group. Although CXCR5^+^PD1^+^ Tfh cells were detectable in the CNS of both groups, their frequencies and absolute numbers remained relatively low. Notably, in the EAE model, substantial CNS infiltration by endogenous Tfh cells occurred exclusively in the Th17 cell adoptive transfer cohort, whereas negligible Tfh infiltration was observed following direct Tfh cell transfer—highlighting fundamental differences in the immunobiology of these two adoptive transfer paradigms (11). Furthermore, flow cytometry data indicated that adoptively transferred B cells alone failed to efficiently cross the blood–brain barrier (BBB) or accumulate within the CNS parenchyma, underscoring their dependence on T-cell-mediated help—particularly via chemokine-directed trafficking and BBB modulation—for pathogenic CNS entry.

Specifically, we note that *Cxcl9* is upregulated in three settings: (i) spinal cord of Tfh-transferred mice, (ii) spinal cord of Th17-transferred mice, and (iii) astrocytes exposed to AQP4-ab alone. This suggests that CXCL9 elevation is not exclusive to T-cell-driven inflammation but can also be induced by direct antibody-mediated astrocyte injury. However, the degree of upregulation and the associated co-expressed gene networks differ between conditions, implying context-dependent roles. We now state that the strong association of CXCL9 with Tfh-, Th17-, and AQP4-ab-mediated responses supports its potential as a common downstream effector or biomarker, rather than a specific mediator of one pathway. While our confocal imaging experiments confirm that astrocytes are capable of expressing CXCL9, additional cellular sources likely contribute to the elevated CXCL9 levels observed in our animal models and in NMOSD patients. In the inflamed CNS, immune cells (T lymphocytes, NK cells), macrophages, and dendritic cells are well-known producers of CXCL9 upon activation (36). Moreover, CNS-resident microglia and vascular endothelial cells could upregulate CXCL9 in response to pro-inflammatory cytokines such as IFN-γ (37). Even within the astrocyte lineage, it is plausible that astrocytes produce CXCL9 at early stages of injury or upon exposure to inflammatory stimuli (e.g., viral mimic or cytokines) before they undergo irreversible damage. In our adoptive transfer models, the transferred AQP4-reactive T cells may directly secrete CXCL9 or induce its expression in recipient cells. Therefore, the elevated CXCL9 detected in spinal cord homogenates likely reflects a composite signal from multiple cellular origins. Dissecting the relative contribution of each source would require future studies using cell-type-specific Cxcl9 reporter or conditional knockout mice, as well as single-cell transcriptomics of NMOSD lesions.

Using quantitative real-time PCR, we observed a significant upregulation of CXCL9 expression in the Tfh- and Th17-transferred groups, consistent with immunohistochemical findings; in contrast, CXCR3 expression exhibited only a modest increase. Chemokines are small, secreted signaling proteins that mediate leukocyte chemotaxis, regulate immune cell differentiation, and facilitate tissue extravasation—processes essential for lymphocyte trafficking into the CNS (37). CXCL9 is a high-affinity ligand for the chemokine receptor CXCR3, which also binds CXCL10 and CXCL11. Under homeostatic conditions, CXCL9 is virtually undetectable; however, its expression is robustly induced during inflammation and infection in an IFN-γ–dependent manner, alongside CXCL10 and CXCL11. This coordinated induction supports the critical role of the CXCR3 axis in recruiting antibody-secreting cells to inflammatory sites (38). Notably, prior clinical studies reported elevated CXCR3 expression on B cells isolated from the cerebrospinal fluid of AQP4-ab-positive NMOSD patients during acute attacks. Moreover, CXCR3 has been identified as the predominant chemokine receptor enabling CNS persistence of B cells, suggesting its functional involvement in peripheral B-cell recruitment across the blood–CNS barrier (39, 40). In a comparative analysis of supernatants from peripheral blood mononuclear cells, Vaknin-Dembinsky et al. demonstrated significantly increased CXCL9 levels—but no significant change in CXCL10—in NMOSD patients relative to healthy controls (21). Collectively, these findings implicate the CXCL9–CXCR3 axis as a key pathogenic pathway in AQP4-ab-positive NMOSD. T-bet (encoded by TBX21), GATA3, and RORC encode lineage-defining transcription factors for Th1, Th2, and Th17 cells, respectively (41). In our model, RORC expression remained unchanged in the Tfh-transferred group, suggesting that Tfh-mediated pathology may occur independently of canonical Th17 differentiation. This observation aligns with reports indicating that Th17 cells can undergo functional plasticity upon transfer into EAE models, acquiring IFN-γ-producing capacity—a shift strongly associated with enhanced pathogenicity (42). Such plasticity may account for the observed upregulation of TBX21 without concomitant RORC induction. Furthermore, adoptive transfer of Th17 cells into EAE mice has been shown to promote acquisition of Tfh-like phenotypic and functional features by a subset of donor-derived T cells (11, 43). The transcriptional regulators BCL-6 and PRDM1 exert mutually antagonistic effects: BCL-6 promotes Tfh cell differentiation and represses PRDM1 expression, whereas PRDM1 inhibits Tfh commitment and drives terminal B-cell differentiation into antibody-secreting plasma cells (44, 45). Given the pronounced B-cell infiltration observed in the CNS of mice receiving Tfh and Th17 cells, it is plausible that infiltrating B cells undergo local differentiation into plasma cells within the CNS parenchyma.

This study has several limitations. First, the cohort size of experimental mice was relatively small, potentially limiting statistical power to detect subtle intergroup differences. Second, the adoptive transfer model—while informative for dissecting T-cell-driven mechanisms—fails to reproduce key features of human AQP4-ab-positive NMOSD, including astrocyte necrosis and complement deposition, and therefore does not fully recapitulate the hallmark humoral and complement-dependent pathology. Third, *in vitro* astrocyte data may not fully recapitulate the *in vivo* microenvironment, and we propose future experiments (e.g., conditional knockout or blocking studies) to dissect the causal role of CXCL9 in each context.

## 5 Conclusions

Collectively, these results confirm that AQP4-reactive Tfh and Th17 cells possess intrinsic encephalitogenic potential. Both models recapitulate hallmark features of NMOSD neuropathology, including glial cell activation, enhanced chemokine-driven leukocyte recruitment, sustained neuroinflammation, focal demyelination, and widespread CNS infiltration by activated myeloid and lymphoid cells. Given its strong association with Tfh- and Th17-driven inflammation and glial cell activation, CXCL9 emerges as a potential contributor for NMOSD.

## Data Availability

The original contributions presented in the study are included in the article/**Supplementary Material**. Further inquiries can be directed to the corresponding authors.
