# Supplementary material for: CXCL9 associates with experimental neuromyelitis optica spectrum disorder following adoptive transfer of Tfh and Th17 cells
Source: Front Immunol. 2026 May 28;17:1704040. doi: 10.3389/fimmu.2026.1704040 (PMC13253477; doi:10.3389/fimmu.2026.1704040)
Supplement: Supplementary file 1 [file DataSheet1.docx]

**Supplementary Figure**

**
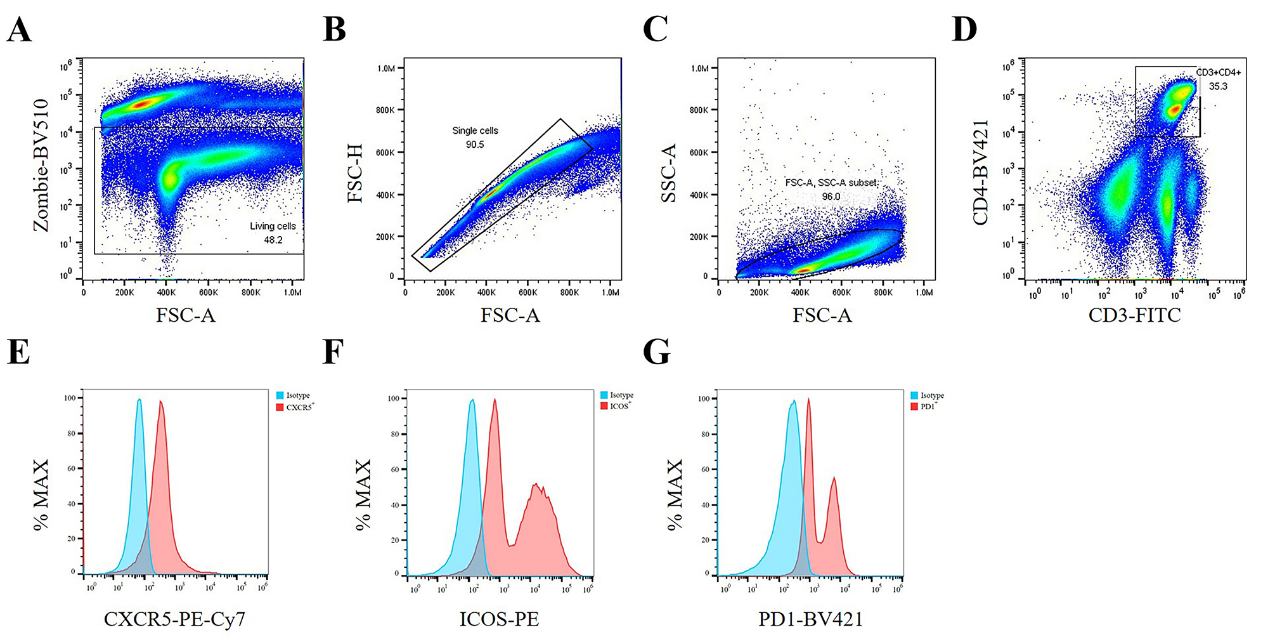
**

**Fig. S1. Gating strategy of Tfh cells from the lymph nodes.** A: Gating viable cells; B: Gating single cells; C: Gating the total lymphocytes; D: Gating CD3^+^CD4^+^ cells from the lymphocyte gate; G: CXCR5 and its isotype from the CD3^+^CD4^+^ cell gate; H: ICOS and its isotype from the CD3^+^CD4^+^ cell gate; I: PD1 and its isotype from the CD3^+^CD4^+^ cell gate.


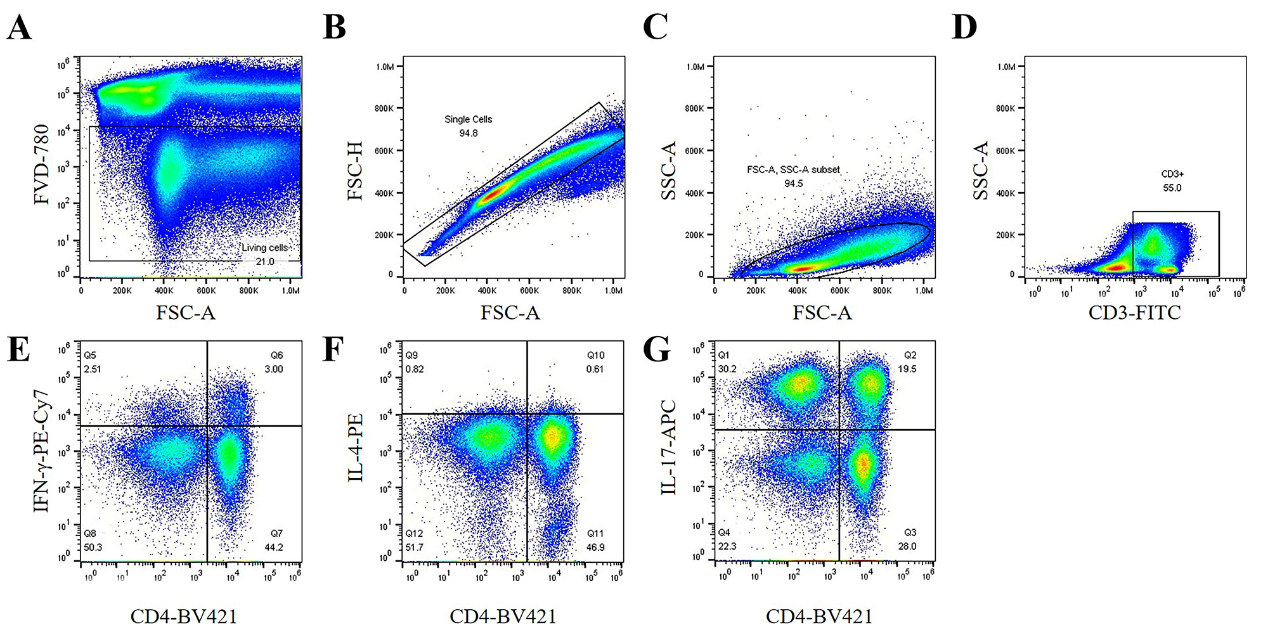


**Fig. S2. Gating strategy of Th17 cells from the lymph nodes.** A: Gating viable cells; B: Gating single cells; C: Gating the total lymphocytes; D: Gating CD3^+^ cells from the lymphocyte gate; E: Draw the cross gate from the CD3^+^ cell gate, CD4^+^IFN-γ^+^ Th1 cells in the upper right quadrant; F: Draw the cross gate from the CD3^+^ cell gate, CD4^+^IL-4^+^ Th2 cells in the upper right quadrant; G: Draw the cross gate from the CD3^+^ cell gate, CD4^+^IL-17^+^ Th17 cells in the upper right quadrant.

**
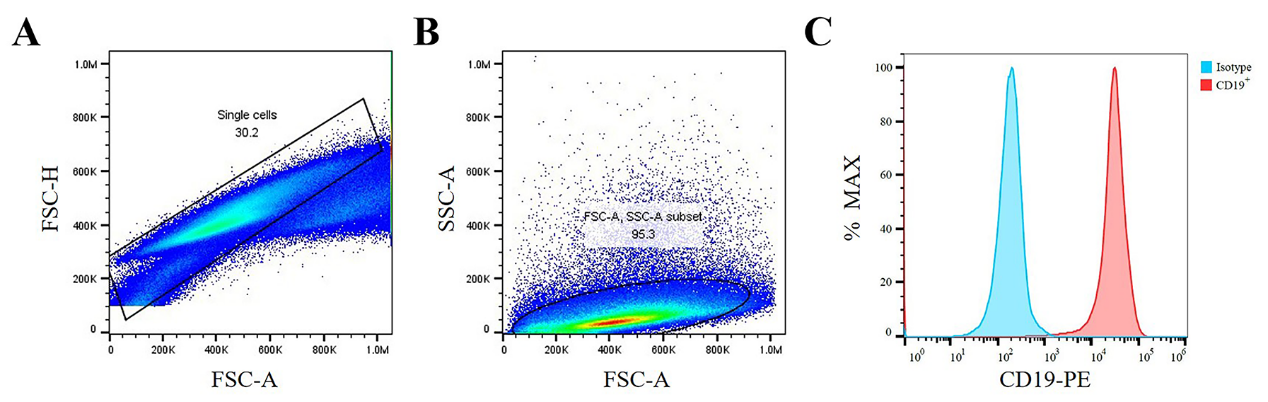
**

**Fig. S3. Gating strategy of B cells from the lymph nodes.** A: Gating single cells; B: Gating the total lymphocytes; C: Gating CD19^+^ cells from the lymphocyte gate.


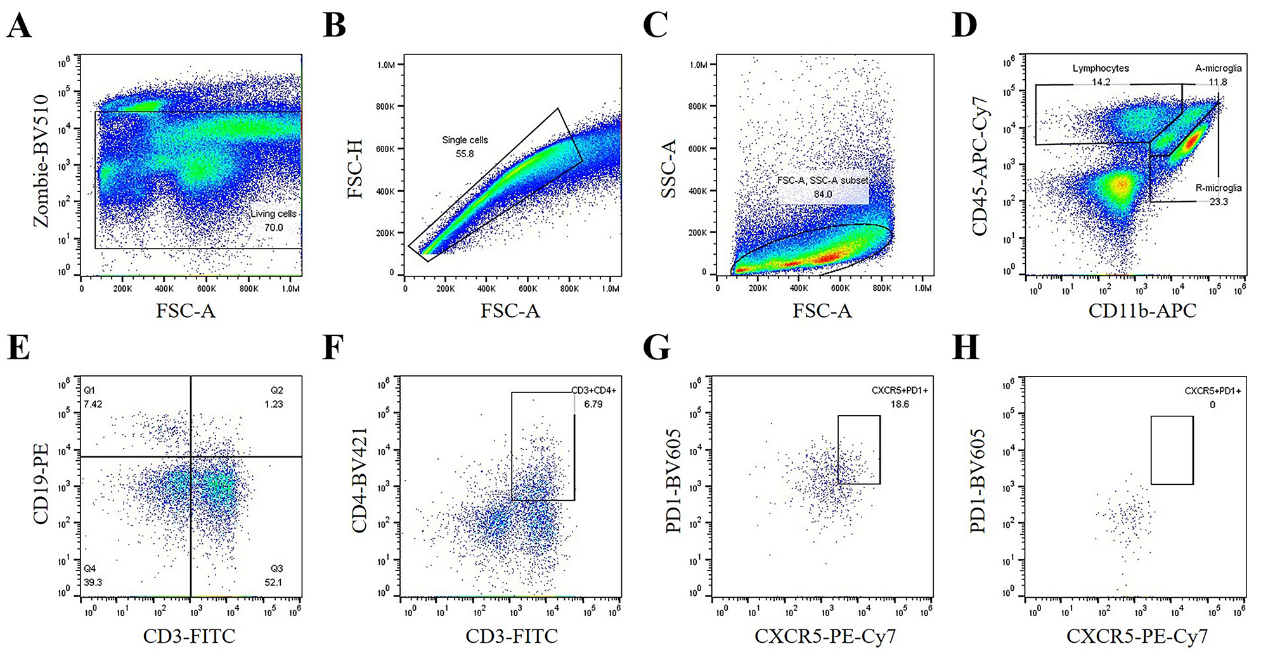


**Fig. S4. Gating strategies of infiltrating lymphocytes subsets and microglia/macrophage in the central nervous system.** A: Gating viable cells; B: Gating single cells; C: Gating the central nervous system cells; D: Gating the CD11b^+^CD45^high^ microglia/macrophage, CD11b^+^CD45^low^ microglia/macrophage and CD11b^-^CD45^+^ lymphocytes; E: Draw the cross gate from the lymphocyte gate, CD3^-^CD19^+^ cells in the upper left quadrant, CD3^-^CD19^-^ cells in the lower left quadrant, and CD3^+^CD19^-^ cells in the lower right quadrant; F: Gating CD3^+^CD4^+^ cells from the lymphocyte gate; G: Gating CXCR5^+^PD1^+^ cells from the CD3^+^CD4^+^ cell gate; H: Isotype control of CXCR5^+^PD1^+^ cells.

**Movie S1. AQP4-reactive Tfh cells cause moderate paraplegia in recipient WT mouse.**

**Movie S2. AQP4-reactive Th17 cells cause severe paraplegia in recipient WT mouse.**
